# Generating prior probabilities for classifiers of brain tumours using belief networks

**DOI:** 10.1186/1472-6947-7-27

**Published:** 2007-09-18

**Authors:** Greg M Reynolds, Andrew C Peet, Theodoros N Arvanitis

**Affiliations:** 1 Department of Electrical, Electronic and Computer Engineering, University of Birmingham, Birmingham, UK; 2 Academic Department of Paediatrics and Child Health, University of Birmingham, UK and Birmingham Children's Hospital NHS Foundation Trust, Birmingham, UK

## Abstract

**Background:**

Numerous methods for classifying brain tumours based on magnetic resonance spectra and imaging have been presented in the last 15 years. Generally, these methods use supervised machine learning to develop a classifier from a database of cases for which the diagnosis is already known. However, little has been published on developing classifiers based on mixed modalities, e.g. combining imaging information with spectroscopy. In this work a method of generating probabilities of tumour class from anatomical location is presented.

**Methods:**

The method of "belief networks" is introduced as a means of generating probabilities that a tumour is any given type. The belief networks are constructed using a database of paediatric tumour cases consisting of data collected over five decades; the problems associated with using this data are discussed. To verify the usefulness of the networks, an application of the method is presented in which prior probabilities were generated and combined with a classification of tumours based solely on MRS data.

**Results:**

Belief networks were constructed from a database of over 1300 cases. These can be used to generate a probability that a tumour is any given type. Networks are presented for astrocytoma grades I and II, astrocytoma grades III and IV, ependymoma, pineoblastoma, primitive neuroectodermal tumour (PNET), germinoma, medulloblastoma, craniopharyngioma and a group representing rare tumours, "other". Using the network to generate prior probabilities for classification improves the accuracy when compared with generating prior probabilities based on class prevalence.

**Conclusion:**

Bayesian belief networks are a simple way of using discrete clinical information to generate probabilities usable in classification. The belief network method can be robust to incomplete datasets. Inclusion of *a priori *knowledge is an effective way of improving classification of brain tumours by non-invasive methods.

## Background

The current "gold standard" for brain tumour diagnosis is histopathology which requires a sample of tumour obtained at operation. These operations have an inherent risk of morbidity and mortality. Magnetic Resonance Imaging (MRI), Magnetic Resonance Spectroscopy (MRS) and other imaging modalities may offer a non-invasive way of making a diagnosis, but no method has yet attained sufficient accuracy to replace histopathology. MRS in particular has been shown to provide useful information about the biochemical content of a brain tumours [1] and numerous methods for classifying brain tumours based on magnetic resonance spectra have been presented [2-6].

When making a classification decision it is intuitively sensible to use as much relevant information as possible, but very few of the published classifiers have attempted to combine information from different modalities and sources (but see [7-10]). This work details a method which uses data from the West Midlands Regional Childhood Tumour Registry (WMRCTR) to produce probabilities of brain tumour class, given its anatomical location.

The WMRCTR provides data from the last five decades on over 1700 childhood cancer patients, mostly in free-text form. During that period the format of the stored data has changed: knowledge of the exact anatomical location has improved with the advent of MRI and the classification scheme for tumours has changed to the WHO [11] system. This presents a considerable challenge to its use in computer-based systems.

The discriminating power of "anatomical location" as a feature for a classifier is not sufficient to make classifications based on this variable alone. However it is envisaged that the probabilities obtained from the WMRCTR data could be used as "informative priors" in existing classification methods. In this work we demonstrate their impact on a simple MRS based classifier. It is worth emphasising that this work focusses on paediatric brain tumours, which are significantly more varied and more difficult to diagnose using MRI alone, than those in adults.

The approach to using the WMRCTR data is based on a graphical representation of Bayesian inference called *belief networks*. Since anatomical location and tumour class are discrete random variables, probabilities can be estimated directly from the data, without the need to rely on assumptions about the form of probability density functions. In the following sections we introduce the belief network method, present some examples and discuss the construction of the final network from the data in the WMRCTR. Finally, the network is presented and demonstrated on some test-cases.

## Methods

### Belief networks

**Figure 1:**
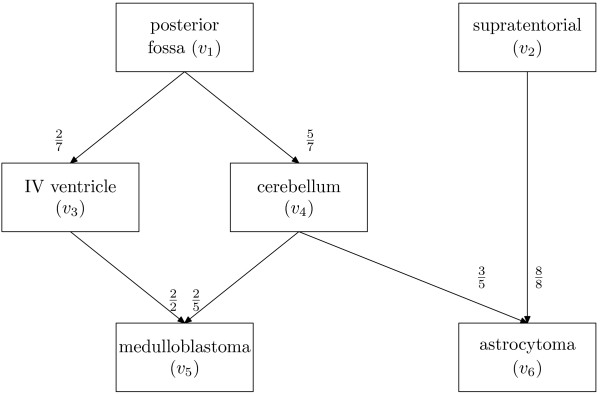
A simplified belief network showing conditioned probabilities of events. The vertex numbers are shown in brackets, refer to the adjacency matrix representation in (1). The numbers shown are purely for pedagogical purposes, for the correct and complete graph refer to Table 1 and Figure 2.

A *Bayesian belief network *or often just *belief network *is a graphical representation of the joint probability distribution function of a collection of variables [12,13]. A belief network makes exactly the same inferences as would be made by applying Bayes' rule to a series of probabilities, but the graphical construction often provides insight into the problem. The network is represented as a weighted, acyclic, directed graph, each vertex representing a discrete variable/event (see Figure 1). To use the terminology of Russell and Norvig [12], each of these vertices fall into one of three categories:

1. *query variables*, i.e. events the probability of which is of interest (in Figure 1 these are "medulloblastoma" and "astrocytoma");

2. *evidence variables*, i.e. events known to have occurred (in Figure 1 these are taken to be "posterior fossa" and "supratentorial");

3. *hidden variables*, i.e. events which may occur but cannot be measured (in Figure 1 these are taken to be "IV ventricle" and "cerebellum").

The weighted edges connecting vertices represent the probability that the target vertex is true, conditioned on the source vertex. Here, vertices represent anatomical locations or tumour types. If an edge connects two anatomical locations then its weight is the probability that the tumour was in the target vertex, given that it is known to be in the source vertex. If an edge connects an anatomical location to a tumour type then its weight is the probability that the tumour is of the type specified by the target vertex, given that it is known to have occurred in the source vertex.

To illustrate the utility of the method, consider the following example. Referring to Figure 1, suppose it is known that the tumour occurs in the posterior fossa (vertex *v*_1_) and the probability that the tumour is a medulloblastoma is sought. Working backward from vertex *v*_5 _(the medulloblastoma) and applying Bayes' rule:

P(medulloblastoma|posterior fossa)=     P(medulloblastoma|IV ventricle)×     P(IV ventricle|posterior fossa)+     P(medulloblastoma|cerebellum)×     P(cerebellum|posterior fossa)=22×27+25×57=47
 MathType@MTEF@5@5@+=feaafiart1ev1aaatCvAUfKttLearuWrP9MDH5MBPbIqV92AaeXatLxBI9gBaebbnrfifHhDYfgasaacH8akY=wiFfYdH8Gipec8Eeeu0xXdbba9frFj0=OqFfea0dXdd9vqai=hGuQ8kuc9pgc9s8qqaq=dirpe0xb9q8qiLsFr0=vr0=vr0dc8meaabaqaciaacaGaaeqabaqabeGadaaakeaafaqaaeGbbaaaaeaacqWGqbaucqGGOaakcqqGTbqBcqqGLbqzcqqGKbazcqqG1bqDcqqGSbaBcqqGSbaBcqqGVbWBcqqGIbGycqqGSbaBcqqGHbqycqqGZbWCcqqG0baDcqqGVbWBcqqGTbqBcqqGHbqycqqG8baFcqqGWbaCcqqGVbWBcqqGZbWCcqqG0baDcqqGLbqzcqqGYbGCcqqGPbqAcqqGVbWBcqqGYbGCcqqGGaaicqqGMbGzcqqGVbWBcqqGZbWCcqqGZbWCcqqGHbqycqGGPaqkcqGH9aqpaeaafaqabeqacaaabaaabaGaaCzcaiaaxMaacaWLjaGaemiuaaLaeiikaGIaeeyBa0MaeeyzauMaeeizaqMaeeyDauNaeeiBaWMaeeiBaWMaee4Ba8MaeeOyaiMaeeiBaWMaeeyyaeMaee4CamNaeeiDaqNaee4Ba8MaeeyBa0MaeeyyaeMaeeiFaWNaeeysaKKaeeOvayLaeeiiaaIaeeODayNaeeyzauMaeeOBa4MaeeiDaqNaeeOCaiNaeeyAaKMaee4yamMaeeiBaWMaeeyzaugaaiabcMcaPiabgEna0cqaauaabeqabiaaaeaaaeaacaWLjaGaaCzcaiaaxMaacqWGqbaucqGGOaakcqqGjbqscqqGwbGvcqqGGaaicqqG2bGDcqqGLbqzcqqGUbGBcqqG0baDcqqGYbGCcqqGPbqAcqqGJbWycqqGSbaBcqqGLbqzcqqG8baFcqqGWbaCcqqGVbWBcqqGZbWCcqqG0baDcqqGLbqzcqqGYbGCcqqGPbqAcqqGVbWBcqqGYbGCcqqGGaaicqqGMbGzcqqGVbWBcqqGZbWCcqqGZbWCcqqGHbqycqGGPaqkcqGHRaWkaaaabaqbaeqabeGaaaqaaaqaaiaaxMaacaWLjaGaaCzcaiabdcfaqjabcIcaOiabb2gaTjabbwgaLjabbsgaKjabbwha1jabbYgaSjabbYgaSjabb+gaVjabbkgaIjabbYgaSjabbggaHjabbohaZjabbsha0jabb+gaVjabb2gaTjabbggaHjabbYha8jabbogaJjabbwgaLjabbkhaYjabbwgaLjabbkgaIjabbwgaLjabbYgaSjabbYgaSjabbwha1jabb2gaTjabcMcaPiabgEna0caaaeaafaqabeqacaaabaaabaGaaCzcaiaaxMaacaWLjaGaemiuaaLaeiikaGIaee4yamMaeeyzauMaeeOCaiNaeeyzauMaeeOyaiMaeeyzauMaeeiBaWMaeeiBaWMaeeyDauNaeeyBa0MaeeiFaWNaeeiCaaNaee4Ba8Maee4CamNaeeiDaqNaeeyzauMaeeOCaiNaeeyAaKMaee4Ba8MaeeOCaiNaeeiiaaIaeeOzayMaee4Ba8Maee4CamNaee4CamNaeeyyaeMaeiykaKIaeyypa0daaaqaamaalaaabaGaeGOmaidabaGaeGOmaidaaiabgEna0oaalaaabaGaeGOmaidabaGaeG4naCdaaiabgUcaRmaalaaabaGaeGOmaidabaGaeGynaudaaiabgEna0oaalaaabaGaeGynaudabaGaeG4naCdaaiabg2da9maalaaabaGaeGinaqdabaGaeG4naCdaaaaaaaa@1538@

Of course, if it was known that the tumour occurred in the IV ventricle then the expression could have stopped there and the probability obtained by inspection, thus hidden variables may sometimes be evidence variables depending on the particular sample. The important point is that different "resolution" information can be used, this is particularly important if a tumour spans several regions, as will be discussed later. It also means that data of lower resolution can be incorporated into the network. For example, many tumours in the WMRCTR are just listed as having location: "posterior fossa". Working back from the query variables is relatively complicated to implement. An easier and equivalent way is to work forward from the evidence variables. As such it is convenient to represent a graph as an *adjacency matrix*, for the example in Figure 1 this is:

A=(002757000000088000022000002535000010000001)
 MathType@MTEF@5@5@+=feaafiart1ev1aaatCvAUfKttLearuWrP9MDH5MBPbIqV92AaeXatLxBI9gBaebbnrfifHhDYfgasaacH8akY=wiFfYdH8Gipec8Eeeu0xXdbba9frFj0=OqFfea0dXdd9vqai=hGuQ8kuc9pgc9s8qqaq=dirpe0xb9q8qiLsFr0=vr0=vr0dc8meaabaqaciaacaGaaeqabaqabeGadaaakeaaieWacqWFbbqqcqGH9aqpdaqadaqaauaabeqagyaaaaaabaGaeGimaadabaGaeGimaadabaWaaSaaaeaacqaIYaGmaeaacqaI3aWnaaaabaWaaSaaaeaacqaI1aqnaeaacqaI3aWnaaaabaGaeGimaadabaGaeGimaadabaGaeGimaadabaGaeGimaadabaGaeGimaadabaGaeGimaadabaGaeGimaadabaWaaSaaaeaacqaI4aaoaeaacqaI4aaoaaaabaGaeGimaadabaGaeGimaadabaGaeGimaadabaGaeGimaadabaWaaSaaaeaacqaIYaGmaeaacqaIYaGmaaaabaGaeGimaadabaGaeGimaadabaGaeGimaadabaGaeGimaadabaGaeGimaadabaWaaSaaaeaacqaIYaGmaeaacqaI1aqnaaaabaWaaSaaaeaacqaIZaWmaeaacqaI1aqnaaaabaGaeGimaadabaGaeGimaadabaGaeGimaadabaGaeGimaadabaGaeGymaedabaGaeGimaadabaGaeGimaadabaGaeGimaadabaGaeGimaadabaGaeGimaadabaGaeGimaadabaGaeGymaedaaaGaayjkaiaawMcaaaaa@5866@

Each element *a*_*ij *_of ***A ***refers to the weighted connection from vertex *v*_*i *_to *v*_*j*_, i.e. the row index refers to the source vertex, the column index to the target.

The adjacency matrix representation permits easy calculation of the vector of class probabilities, given knowledge of which evidence variables to use. To find the probabilities of each class, given any evidence variable the following procedure is used (more computationally efficient methods are given in [12]):

1. Construct the *n*-dimensional column vector ***x ***where *n *is the number of vertices (variables) and set all the elements to zero.

2. Set the single element of ***x ***that corresponds to the evidence variable known be to true, to one.

3. Compute ***x ***← ***A***^*T*^***x ***until ***x ***stops changing. At every iteration, those vertices connected to those with non-zero entries ***x ***will become non-zero.

4. Those elements of ***x ***corresponding to output variables have the probability that the tumour belongs to each class, given the evidence. All other elements of ***x ***will be zero.

Clearly, the axioms of probability require that the sum of all elements in ***x ***is unity. It is important to note that the terminating vertices (*v*_5 _and *v*_6 _in Figure 1) need to be connected to themselves so that the method just described will converge to the correct value. If they are not present, ***x ***will converge to the zero vector. As well as giving probabilities of class membership given a single location, tumours that span adjacent anatomical regions can also be considered; for every region in which the tumour is present compute the output vector, then average these to produce the final vector of probabilities. Although this is an intuitively sensible property, it has not been evaluated in this work.

### Data processing

**Figure 2:**
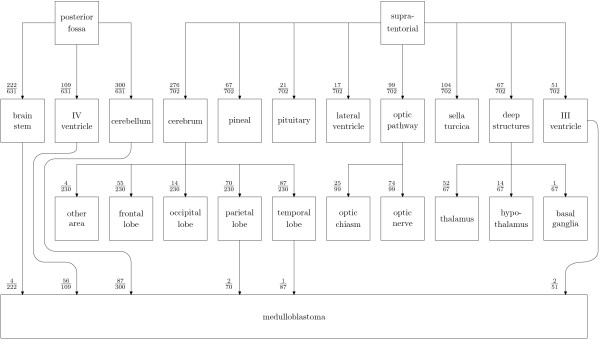
Part of the complete belief network, showing the locations common to all tumour types but just one tumour classification path. The complete specification, including weights and paths for all tumour types covered is shown in Table 1.

The WMRCTR database was made available as a spreadsheet giving hand-typed strings for the diagnosis and location of each case. Occasionally, grade of tumour was also specified. In total there were 1712 cases available. By hand, each record was examined and modified. If the tumour type was classified with the WHO system, it was left unchanged. If a WHO equivalent existed for a tumour classified using the old scheme, then it was changed; otherwise the record was removed. This reduced the number of cases to 1367. These cases were then further reviewed, and only those tumours with location specified were included, reducing the final number of cases used to 1333. Of these, the site of the primary lesion was often only specified vaguely, e.g. "posterior fossa" or "cerebrum". Tumours specified with a greater degree of accuracy were then grouped (by hand) under these broader headings, as well as maintaining their original information. A graph showing the anatomical distinctions made is given in Figure 2. Very occasionally, the location was specified in great detail (e.g. "foramen of Munro") but this was very rare and these samples were marked as being in the appropriate containing location.

The classes used were: astrocytoma grades I and II, astrocytoma grades III and IV, ependymoma, pineoblastoma, PNET, germinoma, medulloblastoma, craniopharyngioma and a group representing rare tumours, "other" (classes represented by fewer than 15 cases). It is common practice to group paediatric astrocytomas of different grades in this way as they thought to be very similar diseases.

## Results and discussion

The simplest method to represent the results would be an adjacency matrix, but this is too large for publication in its direct format. Instead, the graph is specified by Table 1; an *adjacency list *representation. A subgraph of the final belief network is shown in Figure 2, giving the paths necessary to generate a probability for a medulloblastoma.

**Table 1:** Adjacency list representation of final belief network. The destination vertices from each vertex are shown in the "Connections" column

Vertex	Description	Connections (vertex, weight)
*v*_1_	posterior fossa	(*v*_3_, 222/631), (*v*_4_, 109/631), (*v*_5_, 300/631)
*v*_2_	supratentorial	(*v*_6_, 276/702), (*v*_7_, 67/702), (*v*_8_, 21/702), (*v*_9_, 17/702), (*v*_10_, 99/702), (*v*_11_, 104/702), (*v*_12_, 67/702), (*v*_13_, 51/702)
*v*_3_	brain stem	(*v*_24_, 189/222), (*v*_28_, 1/222), (*v*_29_, 3/222), (*v*_30_, 4/222), (*v*_31_, 6/222), (*v*_32_, 19/222)
*v*_4_	IV ventricle	(*v*_24_, 16/109), (*v*_28_, 3/109), (*v*_29_, 24/109), (*v*_30_, 56/109) (*v*_32_, 10/109)
*v*_5_	cerebellum	(*v*_24_, 168/300), (*v*_28_, 9/300), (*v*_29_, 12/300), (*v*_30_, 87/300), (*v*_31_, 3/300), (*v*_32_, 21/300)
*v*_6_	cerebrum	(*v*_14_, 4/230), (*v*_15_, 55/230), (*v*_16_, 14/230), (*v*_17_, 70/230), (*v*_18_, 87/230)
*v*_7_	pineal	(*v*_24_, 3/67), (*v*_26_, 19/67), (*v*_27_, 17/67), (*v*_28_, 1/67) (*v*_32_, 27/67)
*v*_8_	pituitary	(*v*_25_, 12/21), (*v*_32_, 9/21)
*v*_9_	lateral ventricle	(*v*_24_, 5/17), (*v*_29_, 1/17), (*v*_31_, 1/17), (*v*_32_, 10/17)
*v*_10_	optic pathway	(*v*_19_, 25/99), (*v*_20_, 74/99)
*v*_11_	sella turcica	(*v*_24_, 25/104), (*v*_25_, 51/104), (*v*_27_, 10/104), (*v*_28_, 1/104), (*v*_29_, 1/104), (*v*_31_, 1/104), (*v*_32_, 15/104)
*v*_12_	"deep structures"	(*v*_21_, 52/67), (*v*_22_, 14/67), (*v*_23_, 1/67)
*v*_13_	III ventricle	(*v*_24_, 26/51), (*v*_25_, 4/51), (*v*_29_, 3/51), (*v*_30_, 2/51), (*v*_31_, 1/51), (*v*_32_, 15/51)
*v*_14_	"other cerebral area"	(*v*_24_, 3/4), (*v*_32_, 1/4)
*v*_15_	frontal lobe	(*v*_24_, 19/55), (*v*_25_, 3/55), (*v*_28_, 4/55), (*v*_29_, 5/55), (*v*_31_, 8/55), (*v*_32_, 16/55)
*v*_16_	occipital lobe	(*v*_24_, 7/14), (*v*_28_, 2/14), (*v*_29_, 2/14), (*v*_32_, 3/14)
*v*_17_	parietal lobe	(*v*_24_, 37/70), (*v*_28_, 13/70), (*v*_29_, 4/70), (*v*_30_, 2/70), (*v*_31_, 3/70), (*v*_32_, 11/70)
*v*_18_	temporal lobe	(*v*_24_, 51/87), (*v*_28_, 2/87), (*v*_29_, 7/87), (*v*_30_, 1/87), (*v*_31_, 5/87), (*v*_32_, 21/87)
*v*_19_	optical chiasm	(*v*_24_, 22/25), (*v*_25_, 1/25), (*v*_32_, 2/25)
*v*_20_	optic nerve	(*v*_24_, 73/74), (*v*_32_, 1/74)
*v*_21_	thalamus	(*v*_24_, 35/52), (*v*_28_, 1/52), (*v*_29_, 1/52), (*v*_31_, 3/52) (*v*_32_, 12/52)
*v*_22_	hypo-thalamus	(*v*_24_, 10/14), (*v*_27_, 1/14), (*v*_32_, 3/14)
*v*_23_	basal ganglia	(*v*_24_, 1/1)
*v*_24_	astrocytoma G1, G2 and optic pathway glioma	(*v*_24_, 1)
*v*_25_	craniopharyngioma	(*v*_25_, 1)
*v*_26_	pineoblastoma	(*v*_26_, 1)
*v*_27_	germinoma	(*v*_27_, 1)
*v*_28_	PNET	(*v*_28_, 1)
*v*_29_	ependymoma	(*v*_29_, 1)
*v*_30_	medulloblastoma	(*v*_30_, 1)
*v*_31_	astrocytoma G3, G4	(*v*_31_, 1)
*v*_32_	other tumour	(*v*_32_, 1)

The probabilities (weights) for each connection are expressed as a fraction, giving the final quantities obtained from the WMRCTR. For example, 222 cases of the 631 tumours in the posterior fossa, were in the brain stem.

Figure 2 indicates that medulloblastomas have been recorded in the database as occurring in several locations in the brain. Typically however, they are thought to arise from the cerebellum. The IV ventricle and the brain stem are adjacent to the cerebellum and are often invaded by these tumours making it impossible to be certain of the location from which the tumour originated. This illustrates the power of the belief network in dealing with this situation; the possibility of the tumour being a medulloblastoma is not discounted if it does not occur in the typical position.

Figure 2 also illustrates another feature of the network arising from the fact that a large amount of historical data was used: a small number of medulloblastomas were also recorded as being present in the parietal and temporal lobes, which are distant from the cerebellum. These cases may be due to an incorrect assumption being made at the time of diagnosis that a meta-static deposit from the primary medulloblastoma tumour was actually the primary tumour itself. Again this rare but known clinical scenario is well accounted for by the belief network.

To validate the method presented above, two simple classifiers were investigated using data from 46 recent patients forming part of an ongoing study of MRS of childhood brain tumours [14]. Each patient had a tumour from one of seven classes: astrocytoma grade I and II (16 cases, *v*_24_), medulloblastoma (13 cases, *v*_30_), ependymoma (3 cases, *v*_29_), germinoma (3 cases, *v*_27_), PNET (3 cases, *v*_28_), astrocytoma grade III and IV (2 cases, *v*_31_), and "other" (6 cases, *v*_32_).

The first classifier used only the belief network in the classification; assigning to each sample the label of the class with the highest probability as predicted by the network. This classifier had an error rate of 59%, compared with an error rate of 65% when using probabilities predicted by class prevalence.

The second classifier investigated the effect of using the network to augment a basic MRS classifier. Each of the 46 samples available was a short-echo time (30 ms) single voxel spectroscopy acquisition acquired on a Siemens Symphony 1.5T scanner. The free induction decay (FID) contained 1024 points and was sampled at 1000 Hz. Post-acquisition residual water was removed using the HSVD method [15] to model the water component ± 30 Hz either side of the water signal. Each FID was then Fourier transformed with no line-broadening to give the magnitude spectrum and then normalised to have unit length in the *l*_2_-norm. The normalised spectra were feature-reduced using principal components analysis (PCA) to 10 dimensions. Gaussian functions were then used as the discriminant with mean estimated for each tumour class and a common estimate of the covariance matrix shared across all samples. Two scenarios were then investigated, prior probabilities based on class prevalence and prior probabilities using the belief network. Prior probabilities were applied by multiplying the value of each Gaussian discriminant. Classifier performance was measured using three metrics: apparent error, leave-one-out cross-validation error and the 632+ error rate estimator [16].

With prior probabilities based on class prevalence the apparent error rate was 20%, corresponding to a correct classification of 37 out of 46 tumours. However, the cross validation and 632+ error rates were 41% and 48% respectively, indicating a poor generalisation to unseen cases. With prior probabilities based on the belief network the apparent error rate was 15% corresponding to a correct classification of 39 out of 46 tumours, the cross validation and 632+ error rates were 32% and 37% respectively, indicating that the prior probabilities measurably improve the generalisation performance of the classifier. When using belief network prior probabilities, five of the incorrectly classified samples were the same as those incorrectly classified when using class prevalence priors, the remaining two (one ependymoma, one astrocytoma grade I/II) were correctly classified using prevalence information.

The distribution of the error rate among the classes was approximately the same for both methods of generating prior probabilities, although there were slight differences. Complete results are presented in Table 2. In nearly all incorrectly classified cases, the class with the second highest posterior probability was the correct class; although this was true for both methods of generating priors. In the incorrectly classified cases, the difference in posterior probability between the predicted label and the correct label's probability was small (≈ 0.01) for about half the misclassifications and large (≈ 0.4) for the other half; this was also true for both methods of generating prior probabilities.

**Table 2:** Breakdown of Classification Errors

Class	Share of Error (Prevalence)	Share of Error (Belief Network)
astrocytoma G1, G2 *v*_24_	18.5%	15.2%
medulloblastoma *v*_30_	27.1%	25.6%
ependymoma *v*_29_	9.2%	11.3%
germinoma *v*_27_	8.0%	4.8%
PNET *v*_28_	7.6%	7.3%
astrocytoma grade G3, G4 *v*_31_	12.1%	14.1%
other *v*_32_	17.5%	21.6%

Each percentage is the apportionment of total classification errors attributable to each class, obtained over the 920 trials used to estimate the 632+ error.

The simple classifier presented here attempts only to demonstrate that the belief network method can be useful and that the data used for its construction is sufficiently accurate. The application of the belief network to other classifiers depends on the choice of classifier, but many classifiers have a natural way to use prior probabilities either directly or in the form of weights.

## Conclusion

Data from a large clinical database, collected over five decades, was used to construct a Bayesian belief network suitable for generating probabilities of tumour class. The network was shown to enhance a simple probability-based classifier that uses PCA reduced raw MRS spectra for features. It is suggested that additional (discrete) information could be incorporated into the belief network to further enhance classifier performance.

## Competing interests

The author(s) declare that they have no competing interests.

## Authors' contributions

All authors were involved in developing the concept of using prior probabilities in childhood brain tumour classification. GMR constructed the belief networks processed the data and drafted the paper, which was reviewed by TNA and ACP. All authors read and approved the final manuscript.

## Pre-publication history

The pre-publication history for this paper can be accessed here:


